# Topography of Cortical Activation with Mirror Visual Feedback and Electromyography-Triggered Electrical Stimulation: A Functional Near-Infrared Spectroscopy Study in Healthy Older Adults

**DOI:** 10.3390/brainsci15101074

**Published:** 2025-10-02

**Authors:** Yuji Inagaki, Miku Nakatsuka, Yumene Naito, Daisuke Sawamura

**Affiliations:** 1 Department of Rehabilitation Science, Faculty of Health Sciences, Hokkaido University, Sapporo 060-0812, Japan; d.sawamura@pop.med.hokudai.ac.jp; 2 Graduate School of Health Sciences, Hokkaido University, Sapporo 060-0812, Japan; nakatsuka.miku.m5@elms.hokudai.ac.jp; 3 Department of Rehabilitation Science, Faculty of Health Sciences, Kobe University, Kobe 654-0142, Japan; ot-naito@huhp.hokudai.ac.jp; 4 Department of Physiotherapy, Faculty of Medicine, Dentistry and Health Sciences, University of Melbourne, Melbourne, VIC 3053, Australia

**Keywords:** mirror visual feedback, EMG-triggered electrical stimulation, functional near-infrared spectroscopy, aging, neurorehabilitation

## Abstract

Background/Objectives: Stroke often results in lasting upper limb deficits. Mirror visual feedback (MVF) supports motor recovery, and electromyography-triggered electrical stimulation (ES) could enhance engagement. However, the effects in healthy older adults, age-matched to typical patient cohorts, remain insufficiently understood. We tested MVF and MVF + ES using functional near-infrared spectroscopy. Methods: Seventeen right-handed older adults performed left-wrist flexion under three visual conditions: circle fixation, viewing the right hand at rest, and mirror viewing, with/without electrical stimulation to the right-wrist flexors time-locked to left-forearm electromyography. Oxygenated hemoglobin (oxy-Hb) was recorded over the bilateral inferior frontal gyrus (IFG), precentral gyrus (PrG), postcentral gyrus (PoG), supramarginal gyrus (SMG), superior parietal lobule (SPL), and supplementary motor area. Effects were assessed with linear mixed-effects models (stimulation × visual condition); pairwise comparisons of estimated marginal means used Fisher’s least significant difference. Left-forearm electromyography verified comparable effort across conditions. Results: Linear mixed-effects models revealed left-lateralized increases in oxy-Hb, most prominently under mirror viewing with stimulation. Post hoc tests showed high oxy-Hb in the left IFG, PrG, PoG, SMG, and SMA. The left EMG did not differ. Conclusions: In healthy older adults, MVF paired with EMG-triggered ES enhances frontoparietal–motor engagement beyond MVF alone, with recruitment shaped by visuo–proprioceptive congruence. These findings support mechanistic plausibility and motivate dose–response optimization and patient-focused trials testing behavioral transfer in stroke.

## 1. Introduction

Stroke is the leading cause of long-term disability worldwide, with a particularly high incidence in older adults. With an aging global population, the number of stroke survivors living with sequelae continues to increase [1]. More than 50% of stroke survivors experience upper limb motor impairments [2,3], and many patients report persistent dysfunction in the chronic phase [4,5,6]. Upper limb impairment severely restricts the performance of activities of daily living (ADLs), thereby reducing independence and quality of life. Consequently, the development of effective and feasible rehabilitation strategies to restore upper limb function remains an urgent issue.

One such rehabilitation approach for upper limb paresis is mirror therapy, which uses mirror visual feedback (MVF). This method was first introduced by Ramachandran et al. for the treatment of phantom limb pain [7] and was later applied to motor recovery in post-stroke hemiparesis by Altschuler et al. [8]. Subsequently, numerous randomized controlled trials have been conducted, and systematic reviews and meta-analyses have demonstrated its efficacy in improving upper limb function [9,10] and reducing spasticity in patients with stroke [11].

In recent years, novel therapeutic approaches have been developed that combine MVF with techniques, such as electrical stimulation (ES), robotic assistance, and virtual reality in bimanual MVF therapy, aiming to promote afferent input from the affected side and enhance visual feedback [12,13,14]. Zhuang et al. demonstrated that “associated mirror therapy” significantly improved motor function of the paretic upper limb and ADL performance [15]. Furthermore, systematic reviews and meta-analyses have reported that although conventional MVF produces a moderate effect size on paretic upper limb function (standardized mean difference [SMD] = 0.68; 95% confidence interval [CI], 0.34–1.02), hybrid MVF incorporating ES or virtual reality yields a large effect size (SMD = 1.28; 95% CI, 0.89–1.67) [9]. In addition, interventions combining MVF with ES are significantly superior to sham therapy, ES alone, or MVF alone in improving upper limb motor control based on impairment level and gross grasping ability based on activity level [16].

Previous neuroimaging studies, particularly those using functional magnetic resonance imaging (fMRI), have suggested that MVF activates neural networks associated with the mirror neuron system (MNS), including the left inferior frontal gyrus (IFG), superior temporal sulcus, and inferior parietal lobule [17,18]. More recently, studies employing resting-state fMRI have reported that visual input through a mirror facilitates ipsilateral pathway activation toward the paretic upper limb via increased spontaneous activity in the contralesional primary motor cortex (M1) and reconstructs functional connectivity between the bilateral M1, thereby promoting motor signal transmission from the ipsilesional M1 to the contralesional M1 [19]. In addition, MVF recruits the MNS, corrects interhemispheric imbalance, and promotes motor recovery after stroke [20].

In contrast, combined MVF and ES therapy is thought to indirectly contribute to motor recovery in patients with stroke by simultaneously enhancing proprioceptive and tactile inputs, increasing the activation of sensory cortices, and augmenting sensory projections to motor areas [21]. Although recent studies using fMRI, transcranial magnetic stimulation (TMS), and electroencephalography (EEG) have advanced our understanding of the neural mechanisms underlying MVF, the neurophysiological mechanisms underlying combined MVF and ES therapy remain unclear.

In a previous study involving healthy young adults, we examined the effects of mirror visual feedback (MVF) both alone and in combination with electrical stimulation (ES) on brain activity using functional near-infrared spectroscopy (fNIRS) and found that, although mirrored visual input activated the contralateral somatosensory cortex of the nonmoving limb, the addition of ES suppressed cortical activity [22]. In the present study, however, our aim was not to test age-group differences. Rather, our primary objective was methodological: to establish, in a healthy older cohort age-matched to a planned post-stroke study, the feasibility, tolerability, and sensitivity of fNIRS to detect task-dependent modulations induced by MVF, ES, and their combination. Because aging entails changes in brain structure, neurovascular coupling, and sensorimotor processing [23,24], direct validation in older adults is essential instead of extrapolating from young-adult data. Accordingly, we hypothesized that, within healthy older adults, pairing MVF with ES would elicit greater left-lateralized frontoparietal and sensorimotor hemodynamic responses than MVF alone, whereas ES alone would have limited effects. We therefore focused exclusively on an older adult sample and did not include a younger control group; quantitative age-group comparisons are outside the scope of the present methodological validation and will be addressed in subsequent work designed and powered for between-group contrasts. Accordingly, the present study aimed to evaluate the effects of MVF and MVF + ES on upper-limb function and cortical hemodynamics in healthy older adults using fNIRS to clarify their practical utility and underlying neural mechanisms in the age range most relevant to stroke rehabilitation.

## 2. Materials and Methods

### 2.1. Participants

All the participants were volunteers who were confirmed to be free of neurological disorders based on a pre-screening questionnaire. Twenty-two healthy adults (12 men and 10 women; mean age, 63.5 ± 8.6 years) were initially enrolled. All participants were right-handed, as determined by Chapman’s handedness test [25]. Owing to incomplete MRI or fNIRS data, five participants were excluded, leaving a final sample of 17 individuals (nine men and eight women; mean age, 63.4 ± 7.3 years).

This study was approved by the Ethics Committee of Hokkaido University Hospital on 5 September 2012 (Clinical Research No. 012-0086). Although more than a decade has passed since the original approval, the data have been securely preserved and anonymized, and the current manuscript is based solely on secondary analysis of those previously collected data. Therefore, the ethical approval remains valid for this study. All the participants received a detailed explanation of the study objectives, ES and fNIRS procedures, associated safety considerations, potential risks, and their management. Written informed consent was obtained from each participant before enrollment.

### 2.2. Experimental Overview

#### 2.2.1. Experimental Condition Setup

The participants were seated in a reclining chair, and a mirror box was placed on a horizontal table in front of them. They were instructed to insert both hands into the mirror box with their forearms in a pronated position (ulnar side down). The participants were also instructed to remove accessories, such as rings and watches. Room temperature was maintained at 24 °C.

#### 2.2.2. Tasks

**Figure 1:**
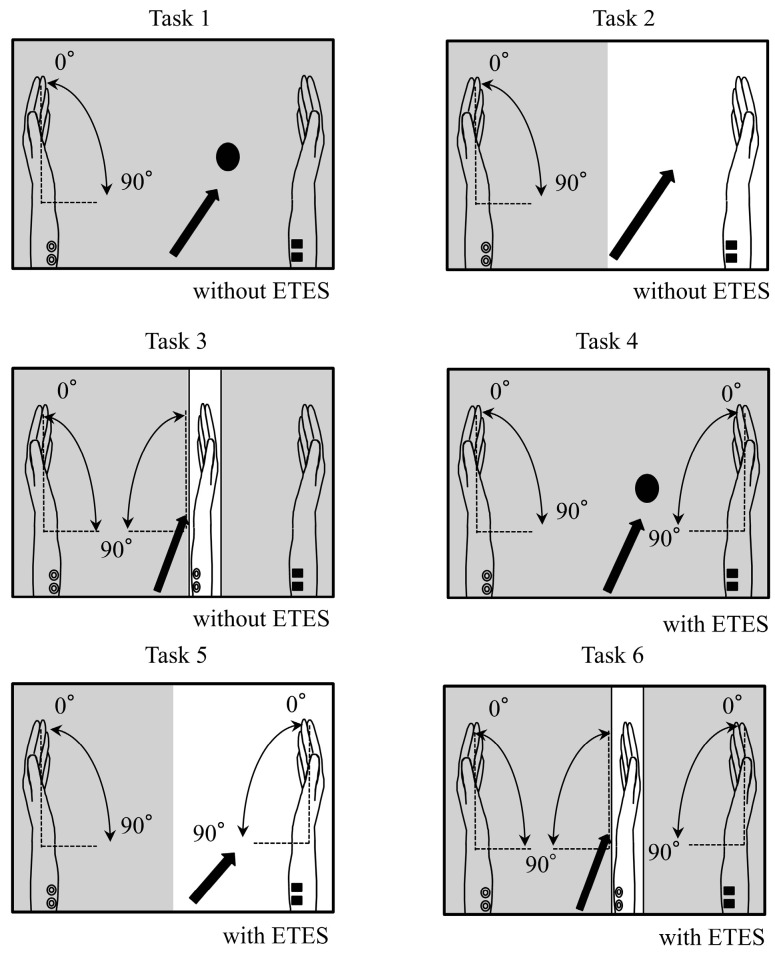
Task conditions. In all conditions, participants performed left-wrist flexion. Arrows indicate the instructed gaze point, and shaded areas are occluded from view. The right hand was still in Tasks 1–3 and moved only via electrical stimulation in Tasks 4–6. In mirror conditions (Tasks 3 and 6), the mirror image of the moving left hand visually overlapped the right hand. The mirror-box lid was closed in Tasks 1 and 4 and open in Tasks 2, 3, 5, and 6. Panels: Task 1—Circle fixation, no stimulation; Task 2—Right hand at rest, no stimulation; Task 3—Mirror viewing, no stimulation; Task 4—Circle fixation with electrically induced right-wrist flexion; Task 5—Right hand with electrically induced right-wrist flexion; Task 6—Mirror viewing with electrically induced right-wrist flexion.

The tasks used in the experiment are illustrated in Figure 1. In all tasks, participants performed repetitive left-wrist flexion at 0.5 Hz for 30 s. A metronome (Art Metronome, Mu-tech Co., Inc, Nara, Japan) was used during both practice and the main experiment to control pacing. Adherence to the 0.5 Hz rhythm was monitored by the experimenter throughout the session.

Before the main experiment, participants practiced while receiving electromyography-triggered electrical stimulation to the right wrist flexors, with the trigger derived from left forearm activity, so that the induced right-wrist movement occurred in synchrony with voluntary left-wrist flexion. During the experiment, participants were instructed to keep the right upper limb relaxed in all conditions. In Tasks 1–3, the right hand was kept completely still. In Tasks 4–6, right-hand motion was produced only by electrical stimulation; participants were explicitly instructed not to move the right hand voluntarily.

The six conditions are illustrated in Figure 1:

Task 1 (Circle, no stimulation):

The lid of the mirror box was closed. Participants fixated on a black circle on the lid and could not see their hands.

Task 2 (Right hand at rest, no stimulation):

The lid was open. Participants observed their right hand at rest; the hand remained completely still throughout this task.

Task 3 (Mirror, no stimulation):

The right hand was placed behind a mirror inside the box. Participants viewed the mirror image so that the moving left hand visually overlapped the right hand.

Task 4 (Circle, stimulation):

Right-wrist flexion was electrically induced in synchrony with left-wrist flexion while participants fixated on the black circle with the lid closed.

Task 5 (Right hand with stimulation):

Participants observed the electrically induced movement of the right hand.

Task 6 (Mirror with stimulation):

As in Task 3, but with electrically induced right-hand movement.

To address movement similarity between the hands: the timing of right-wrist movement matched the left-wrist flexion because the stimulation was triggered by left forearm activity. We did not instruct finger extension; thus, the fingers typically remained flexed while the wrist extended under stimulation. No kinematic instrumentation of the right wrist was used; similarity was enforced by the trigger linkage and monitored by the experimenter.

To verify that the activity of the left forearm wrist flexors remained consistent, electromyographic (EMG) electrodes were attached between the medial epicondyle of the humerus and the palmar base of the second metacarpal, at a site 3–10 cm distal to the medial epicondyle (corresponding to the belly of the muscle), using disposable electrodes (Vitrode F; Nihon Kohden Corp., Tokyo, Japan). The EMG signals were connected directly to a multichannel amplifier (MEG-6108M, Nihon Kohden Co., Ltd., Kyoto, Japan) and monitored in real time using the fNIRS system.

#### 2.2.3. Electromyography-Triggered Electrical Stimulation

The ETES system used in our previous study [22] was used. The stimulation electrodes (rubber electrodes) were placed 2–3 cm apart on the right forearm flexor muscles, and the triggering EMG was recorded from the left flexor carpi radialis using surface-disposable Ag-AgCl electrodes (Vitrode F, Nihon Kohden, Tokyo, Japan). The ground electrode was placed on the left olecranon. This method, developed by Futami et al. [26], enables right hand movement to be elicited by voluntary left-hand movement. In ETES, ES is triggered by EMG activity.

The stimulation parameters were as follows: frequency, 20 Hz; and pulse width, 500 ms. The ES intensity was set below each participant’s pain threshold but above the motor threshold before task initiation. The sensitivity of the triggered EMG was adjusted so that full joint movements could be elicited by ES.

#### 2.2.4. Functional Near-Infrared Spectroscopy Settings

**Figure 2:**
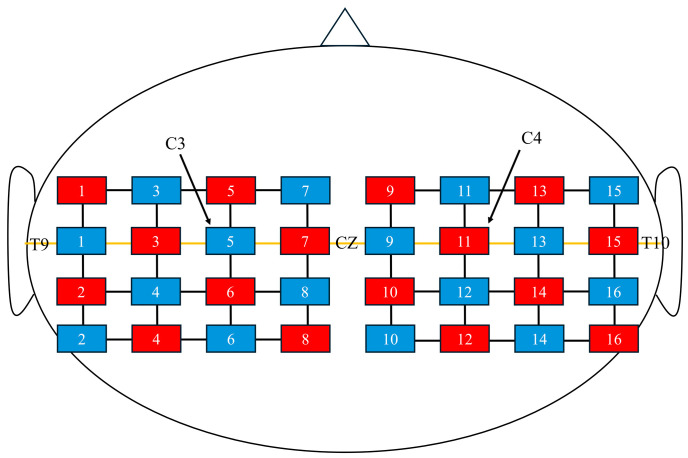
Near-infrared spectroscopy channel configuration. Red squares represent optical sources, and blue squares represent detectors. The midline (orange line) corresponds to the T9–Cz–T10 line. C3 and C4 indicate the standard 10–20 system electrode positions for reference.

Hemodynamic responses were measured using fNIRS (FOIRE 3000; Shimadzu Co., Ltd., Kyoto, Japan) with a holder consisting of 16 light-source probes and 16 detector probes arranged in a 4 × 4 × 2 configuration, yielding 48 channels. The probe placement was based on the virtual registration method described by Tsuzuki et al. [27]. Specifically, the second column of probes from the front was aligned with the coronal reference curve, and the probe located in the second row from the top and second column from the front was positioned at C3 (or C4 for the right hemisphere) according to the international 10–20 system [28] (Figure 2). The distance between the light-source and detector probes was set at 3 cm.

Three wavelengths (708, 805, and 830 nm) were used to detect oxygenated hemoglobin (oxy-Hb), deoxygenated hemoglobin (deoxy-Hb), and total hemoglobin (total-Hb). A three-dimensional (3D) digitizer (FASTRAK; Polhemus, Colchester, VT, USA) was used to determine the anatomical location of each channel. In addition, 3D T1-weighted MR images of all participants were acquired using an MRI scanner (Signa Lightning; GE Healthcare). The channel positions obtained from the 3D digitizer were co-registered with each participant’s cortical surface using NIRS-SPM [29]. The co-registered channels were classified into the bilateral IFG, precentral gyrus (PrG), postcentral gyrus (PoG), supplementary motor area (SMA), supramarginal gyrus (SMG), and superior parietal lobule (SPL).

Because the spatial resolution of fNIRS is limited to approximately 2–3 cm [30], channels located within the sulci were excluded from the analysis. To standardize the changes in oxy-Hb levels, we confirmed the stability of the oxy-Hb signals at the onset of each task.

### 2.3. Data Processing

Based on previous reports indicating that changes in oxy-Hb concentration are known to be more sensitive to neural activity than changes in deoxy-Hb or total-Hb concentration [31,32,33], the present study focused on analyzing oxy-Hb concentration changes. Oxy-Hb signals were obtained using the modified Beer–Lambert law [34] implemented in the fNIRS system. Baseline correction was performed by setting the mean concentration change during the 5 s preceding the task onset to zero. Motion artifacts were corrected using spline interpolation. The raw optical density signals were band-pass filtered (0.01–0.2 Hz) to attenuate slow drifts and physiological noise (e.g., respiration and heartbeat).

Because oxy-Hb responses to neural activity occur with a delay after task onset [35], the mean oxy-Hb concentration was calculated over a 25 s window from 5 to 30 s after task initiation.

The mean oxy-Hb values obtained from each task were analyzed using linear mixed-effects models (LMMs), in which subjects were treated as random effects and Task (visual condition: circle fixation, right hand, mirror) and electrical stimulation (with or without ES) were modeled as within-subject fixed factors. This approach enabled the inclusion of data with missing channels (missing at random) and accounted for the nested structure of repeated measurements within participants. Analyses were conducted separately for each region of interest (ROI).

Post hoc pairwise comparisons were conducted using the least significant difference (LSD) method. Although LSD does not control the familywise error rate as strictly as Bonferroni, it was adopted here as an exploratory approach to retain statistical power given the relatively small sample size and the exploratory nature of this study. Statistical significance was set at *p* < 0.05. All statistical analyses were performed using SPSS version 30 for Windows (SPSS Inc., Chicago, IL, USA).

For EMG, signals from the left-wrist flexors were recorded at a sampling rate of 1000 Hz, band-pass filtered between 20 and 450 Hz, and full-wave-rectified. EMG amplitude was quantified as the root mean square value over the 25 s task window (5–30 s after task onset). These values were analyzed using the same LMM framework to verify that muscle activation of the left forearm did not differ significantly between tasks.

## 3. Results

LMM analyses were conducted with visual condition and ES condition as fixed factors and subjects as random effects for each ROI. Post hoc pairwise comparisons were performed using the LSD method (Table 1).

Left IFG: A significant main effect of Task was observed. Post hoc analysis revealed significantly higher oxy-Hb concentrations in Task 5 compared with Task 3 (β = 0.009, 95% CI [0.003, 0.016], t(71) = 2.80, *p* = 0.006) and in Task 6 compared with Task 3 (β = 0.010, 95% CI [0.003, 0.016], t(71) = 3.04, *p* = 0.003).

Left PoG: Post hoc analysis revealed a significantly higher oxy-Hb concentration in Task 6 compared with Task 1 (β = 0.006, 95% CI [0.000, 0.011], t(96) = 2.09, *p* = 0.039).

Left PrG: Post hoc analyses demonstrated significantly higher oxy-Hb concentrations in Task 6 compared with Task 1 (β = 0.004, 95% CI [0.000, 0.008], t(96) = 2.00, *p* = 0.049), and Task 6 compared with Task 3 (β = 0.004, 95% CI [0.000, 0.008], t(96) = 2.03, *p* = 0.046).

Left SMA: Post hoc analysis revealed significantly higher oxy-Hb concentrations in Task 6 compared with Task 1 (β = 0.006, 95% CI [0.000, 0.011], t(96) = 2.13, *p* = 0.035).

Left SMG: Post hoc analyses revealed significantly higher oxy-Hb concentrations in Task 6 compared with Task 1 (β = 0.006, 95% CI [0.000, 0.012], t(96) = 2.07, *p* = 0.041) and in Task 6 compared with Task 3 (β = 0.006, 95% CI [0.001, 0.012], t(96) = 2.16, *p* = 0.033).

**Figure 3:**
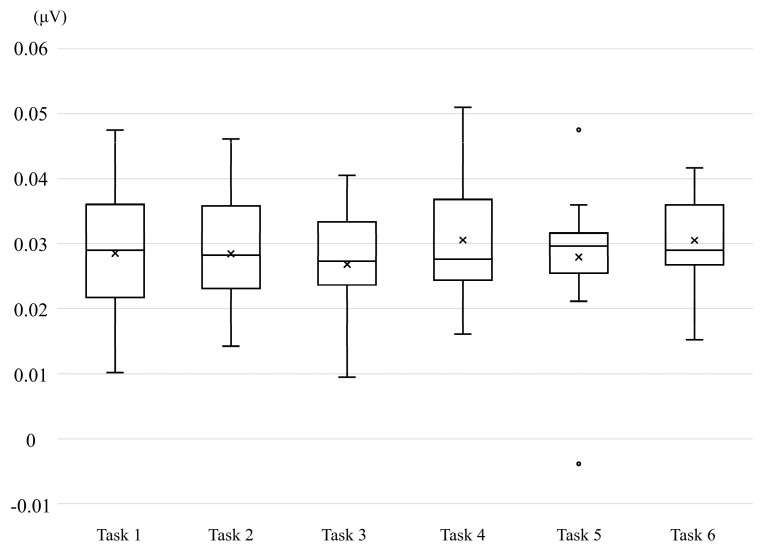
Box-and-whisker plot of electromyographic activity recorded from the left forearm.

Finally, no significant differences were observed in the EMG amplitudes of the left forearm (Figure 3).

## 4. Discussion

This study examined how pairing mirror visual feedback with peripheral electrical stimulation modulates cortical hemodynamics in healthy older adults. Linear mixed-effects models revealed a left-lateralized facilitation pattern, with the most robust increases under mirror viewing with stimulation (Task 6). Post hoc contrasts localized these effects to specific regions: left IFG showed higher oxy-Hb in Task 5 > Task 3 and Task 6 > Task 3; left PrG in Task 6 > Task 1 and Task 6 > Task 3; left PoG in Task 6 > Task 1; SMG in Task 6 > Task 1 and Task 6 > Task 3; and left SMA in Task 6 > Task 1. These findings indicate that electrical stimulation generally amplifies the frontoparietal and sensorimotor responses engaged by mirror-based action observation, with the combination of mirror viewing and synchronized right-wrist activation producing the strongest modulation. Importantly, left-forearm electromyography did not differ across conditions, supporting that the hemodynamic differences were not driven by between-condition effort.

PoG corresponds to the primary somatosensory cortex and is known to respond selectively to tactile and proprioceptive afferent input [36]. In the present study, neither ES alone (Task 4) nor observation of an electrically driven right hand (Task 5) produced a significant increase relative to the control (Task 1). Conversely, under mirror viewing with ES (Task 6), PoG was significantly increased compared with Task 1. Prior work has shown that visually confirming the stimulated body part can enhance tactile discrimination and upregulate S1 activity [37], and EEG studies indicate that observation of biologically plausible, smooth movements strengthens μ-rhythm event-related desynchronization, whereas kinematically unnatural movements attenuate it [38]. The electrically induced movement in Task 5 is unlikely to have been perceived as a “natural self-movement,” which may account for the limited enhancement of PoG. In contrast, during mirror viewing (Task 6), afferent input and visually conveyed information of a “natural” movement through the mirror were likely integrated in a consistent manner.

In contrast, in Task 6, oxy-Hb in the IFG, SMG, SMA, and PrG increased significantly compared with Task 3. This pattern accords with the interpretation that afferent somatosensory input generated by ES is integrated with the MNS recruited by action observation (IFG/SMG), thereby amplifying activity across the frontoparietal–motor network [38,39]. Notably, the IFG increase observed in Task 5 suggests that the simultaneous presentation of observation and afferent input can enhance integrative processing in IFG even without a mirror. However, the more extensive enhancement encompassing SMG/PrG was most pronounced under mirror viewing with ES, indicating that the visual–proprioceptive congruence afforded by mirror viewing preferentially strengthened the mapping from the MNS to primary motor circuitry. Clinically, motor resonance can be elicited even in patients with severe upper-limb impairment, reinforcing the rationale for observation-based protocols as a viable route to engage frontoparietal-motor circuits after stroke [40].

As a supplementary observation, PrG was higher in Task 6 than in Task 1 and Task 3, suggesting increased excitability of corticospinal neurons. In a TMS study of patients with stroke, Zhang et al. reported that motor-evoked potentials in M1 increased only after MVF [41], and the Task-6–related increase in PrG oxy-Hb in the present study is directionally consistent with that finding. However, because motor-evoked potentials were not measured here, a direct test of corticospinal excitability remains a goal for future work. Furthermore, SMA was higher in Task 6 than in Task 1, indicating greater demands on motor preparation/sequence processing under visuo-somatosensory congruence; yet the difference from Task 3 was not pronounced, suggesting that SMA may be more susceptible to afferent input driven by ES than to mirror-specific effects [42].

Unlike in our previous study [22], the ES tasks in this study did not reduce oxy-Hb concentration but instead increased it across the hemisphere corresponding to the stimulated muscles. Guirro et al. investigated the differences in sensory and motor thresholds for transcutaneous ES with respect to sex and age [43]. They found that motor thresholds were higher in older adults. Because older adults tend to have a higher fat-to-muscle ratio, the current resistance is likely to increase, which in turn may necessitate a greater stimulation intensity to excite the motor nerves. This suggests that age-related changes in tissue composition are related to increased threshold intensity. In the present study, the ES intensity was set above the motor threshold but below the pain threshold. However, because the actual output levels were not measured, it remains unclear whether interindividual differences exist. However, this issue requires further investigation.

First, the sample comprised healthy older adults who were all right-handed and performed a simple wrist-flexion task; therefore, caution is warranted when generalizing to patients with stroke or to more complex, ADL-like movements. Second, the final analysis included only 17 participants, and the number of analyzable observations varied across ROIs (e.g., right IFG), which introduces uncertainty in effect estimation and raises the risk of Type II error. A priori sample size estimation using GPower, based on a repeated-measures ANOVA with within-subject factors (effect size f = 0.25, α = 0.05, power = 0.80, 3 × 2 design), indicated that approximately 18–24 participants would be required. Allowing for an estimated 20% dropout rate, a target enrollment of about 22–29 participants would be advisable for future studies. Third, fNIRS captures signals from superficial cortex and cannot fully eliminate contamination from skin blood flow or systemic physiological noise. Moreover, because all conclusions rely exclusively on ROI-wise oxy-Hb changes, which index hemodynamics rather than neuronal firing, claims about recruitment of specific networks and any clinical implications should be considered indirect and provisional unless supported by converging measures (deoxy-/total-Hb, short-separation regression, EEG/TMS). Fourth, although stimulation intensity was titrated individually to “above motor threshold and below pain threshold,” actual device parameters (e.g., current amplitude, pulse width, total charge) and subjective intensity ratings were not recorded, limiting control over inter-individual variability in afferent input. Fifth, regarding the LMM, the results may depend on modeling choices (e.g., random-effects structure, covariance specification, and degrees-of-freedom estimation), assumptions (normality and homoscedasticity of residuals), and the “missing at random” assumption used to accommodate channels/ROIs with missing data. Given the modest sample size and unbalanced cells across ROIs, variance-component estimates and fixed-effect standard errors can be unstable, and small deviations from assumptions (e.g., influential subjects, heteroskedastic residuals across conditions) may disproportionately affect inference. Sixth, multiple ROI-wise tests and post hoc pairwise comparisons increase the family-wise Type I error rate; the use of LSD pairwise tests, while sensitive, provides limited protection against multiplicity, and the findings should be interpreted with this inflation risk in mind. Seventh, corticospinal excitability was inferred indirectly from hemodynamics; without TMS/MEP or other neurophysiological measures, causal claims about M1 output should be considered provisional and tested in future studies.

## 5. Conclusions

In healthy older adults, combining MVF with EMG-triggered ES augments engagement of frontoparietal and sensorimotor systems, supporting the mechanistic plausibility of hybrid MVF + ES protocols that are gaining traction in neurorehabilitation. Beyond demonstrating feasibility with fNIRS in an age group most relevant to stroke, the present work identifies visual context as a tunable design variable: the degree of visuo–proprioceptive congruence appears to shape both the locus and magnitude of cortical recruitment. These insights encourage translational efforts aimed at optimizing visual feedback and afferent drive to promote motor recovery. Future directions include: (i) dose–response studies that parameterize ES (e.g., intensity, pulse width, total charge, and timing relative to voluntary EMG) and delineate its interactions with mirror-based visual cues; (ii) multimodal mechanistic assays, combining fNIRS with TMS and EEG, together with kinematic instrumentation and perceptual ratings of agency/naturalness, to test whether hemodynamic changes track corticospinal excitability and behavior; (iii) patient-focused trials evaluating functional transfer across impairment, activity, and ADL outcomes, identifying responders, and testing whether tailoring the visual context (mirror vs. direct observation) enhances efficacy across lesion profiles and recovery stages; and (iv) larger samples and model-based channel-level analyses with robust control of multiplicity and missingness to refine target ROIs and develop practical biomarkers for individualized MVF + ES prescription in routine care.

## Figures and Tables

**Table 1:** Pairwise task comparisons of oxygenated hemoglobin by region of interest from linear mixed-effects models (Fisher’s LSD).

Region of Interest	Task	Estimate	SE	*df*	95% CI		*t*	*p*
					Lower	Upper		
Left IFG	Task5 > Task3	0.009	0.003	71	0.003	0.016	2.8	0.006
	Task6 > Task3	0.01	0.003	71	0.003	0.016	3.04	0.003
Left PoG	Task6 > Task1	0.006	0.003	96	0.000	0.008	2.09	0.039
Left PrG	Task6 > Task1	0.004	0.002	96	0.000	0.008	2.00	0.049
	Task6 > Task3	0.004	0.002	96	0.000	0.008	2.03	0.046
Left SMA	Task6 > Task1	0.006	0.003	96	0.000	0.011	2.13	0.035
Left SMG	Task6 > Task1	0.006	0.003	96	0.000	0.012	2.07	0.041
	Task6 > Task3	0.006	0.003	96	0.001	0.012	2.16	0.033

IFG: inferior frontal gyrus, PoG: postcentral gyrus, PrG: precentral gyrus, SMA: supplementary motor area, SMG: supramarginal gyrus, SE: standard error, *df*: degrees of freedom, CI: confidence interval.

## Data Availability

The data supporting the findings of this study are available from the corresponding author upon reasonable request. The data are not publicly available because of privacy and ethical restrictions.

## Abbreviations

The following abbreviations are used in this manuscript:

ADL	Activities of daily living
MVF	Mirror visual feedback
ES	Electrical stimulation
SMD	Standardized mean difference
CI	Confidence interval
fMRI	Functional magnetic resonance imaging
MNS	Mirror neuron system
IFG	Inferior frontal gyrus
M1	Primary motor cortex
TMS	Transcranial magnetic stimulation
EEG	Electroencephalography
fNIRS	Functional near-infrared spectroscopy
ETES	EMG-triggered electrical stimulation
EMG	Electromyographic
oxy-Hb	Oxygenated hemoglobin
deoxy-Hb	Deoxygenated hemoglobin
total-Hb	Total hemoglobin
PrG	Precentral gyrus
PoG	Postcentral gyrus
SMG	Supramarginal gyrus
SMA	Supplementary motor area
SPL	Superior parietal lobule
LMMs	linear mixed-effects models
LSD	least significant difference
ROI	Region of interest

## References

[1] GBD 2021 Stroke Risk Factor Collaborators (2024). Global, regional, and national burden of stroke and its risk factors, 1990–2021: A systematic analysis for the Global Burden of Disease Study 2021. Lancet Neurol..

[2] Jørgensen H.S., Nakayama H., Raaschou H.O., Vive-Larsen J., Støier M., Olsen T.S. (1995). Outcome and time course of recovery in stroke. Part I: Outcome. The Copenhagen Stroke Study. Arch. Phys. Med. Rehabil..

[3] Lawrence E.S., Coshall C., Dundas R., Stewart J., Rudd A.G., Howard R., Wolfe C.D. (2001). Estimates of the prevalence of acute stroke impairments and disability in a multiethnic population. Stroke.

[4] Bernardo-Filho M., Bemben M.G., Taiar R., Sañudo B., Furness T., Clark B.C. (2020). Editorial: Interventional strategies for enhancing quality of life and health span in older adults. Front. Aging Neurosci..

[5] Xue S., Zhou X., Yang Z.H., Si X.K., Sun X. (2023). Stroke-induced damage on the blood-brain barrier. Front. Neurol..

[6] Lekoubou A., Nguyen C., Kwon M., Nyalundja A.D., Agrawal A. (2023). Post-stroke everything. Curr. Neurol. Neurosci. Rep..

[7] Ramachandran V.S., Rogers-Ramachandran D. (1996). Synaesthesia in phantom limbs induced with mirrors. Proc. Biol. Sci..

[8] Altschuler E.L., Wisdom S.B., Stone L., Foster C., Galasko D., Llewellyn D.M., Ramachandran V.S. (1999). Rehabilitation of hemiparesis after stroke with a mirror. Lancet.

[9] Kim H., Lee E., Jung J., Lee S. (2023). Utilization of mirror visual feedback for upper limb function in poststroke patients: A systematic review and meta-analysis. Vision.

[10] Zeng W., Guo Y., Wu G., Liu X., Fang Q. (2017). Mirror therapy for motor function of the upper extremity in patients with stroke: A meta-analysis. J. Rehabil. Med..

[11] Muñoz-Gómez E., Inglés M., Aguilar-Rodríguez M., Sempere-Rubio N., Mollà-Casanova S., Serra-Año P. (2023). Effects of mirror therapy on spasticity and sensory impairment after stroke: Systematic review and meta-analysis. PM R.

[12] Wei X., Zhang W., Zhang X., Sui Y., Yu W., Yuan Y. (2023). Effects of contralateral controlled functional electrical stimulation combined with mirror therapy on motor recovery and negative mood in stroke patients. Am. J. Transl. Res..

[13] Qian J., Liang C., Liu R., Yu J., Yang T., Bai D. (2025). Combination of robot-assisted glove and mirror therapy improves upper limb motor function in subacute stroke patients: A randomized controlled pilot study. Front. Neurol..

[14] Choi H.S., Shin W.S., Bang D.H. (2019). Mirror therapy using gesture recognition for upper limb function, neck discomfort, and quality of life after chronic stroke: A single-blind randomized controlled trial. Med. Sci. Monit..

[15] Zhuang J.Y., Ding L., Shu B.B., Chen D., Jia J. (2021). Associated mirror therapy enhances motor recovery of the upper extremity and daily function after stroke: A randomized control study. Neural Plast..

[16] Gurbuz N., Ikbali Afsar S., Ayaş S., Saracgil Cosar S.N. (2016). Effect of mirror therapy on upper extremity motor function in stroke patients: A randomized controlled trial. J. Phys. Ther. Sci..

[17] Yavuzer G., Selles R., Sezer N., Sutbeyaz S., Bussmann J.B., Koseoglu F., Atay M.B., Stam H.J. (2008). Mirror therapy improves hand function in subacute stroke: A randomized controlled trial. Arch. Phys. Med. Rehabil..

[18] Matthys K., Smits M., van der Geest J.N., van der Lugt A., Seurinck R., Stam H.J., Selles R.W. (2009). Mirror-induced visual illusion of hand movements: A functional magnetic resonance imaging study. Arch. Phys. Med. Rehabil..

[19] Zhang K., Ding L., Wang X., Zhuang J., Tong S., Jia J., Guo X. (2024). Evidence of mirror therapy for recruitment of ipsilateral motor pathways in stroke recovery: A resting fMRI study. Neurotherapeutics.

[20] Zhang J.J.Q., Fong K.N.K., Welage N., Liu K.P.Y. (2018). The activation of the mirror neuron system during action observation and action execution with mirror visual feedback in stroke: A systematic review. Neural Plast..

[21] Pan H., Liu T.W., Ng S.S.M., Chen P.M., Chung R.C.K., Lam S.S.L., Li C.S.K., Chan C.C.C., Lai C.W.K., Ng W.W.L. (2024). Effects of mirror therapy with electrical stimulation for upper limb recovery in people with stroke: A systematic review and meta-analysis. Disabil. Rehabil..

[22] Inagaki Y., Seki K., Makino H., Matsuo Y., Miyamoto T., Ikoma K. (2019). Exploring hemodynamic responses using mirror visual feedback with electromyogram-triggered stimulation and functional near-infrared spectroscopy. Front. Hum. Neurosci..

[23] Cabeza R. (2002). Hemispheric asymmetry reduction in older adults: The HAROLD model. Psychol. Aging.

[24] Brodoehl S., Klingner C., Stieglitz K., Witte O.W. (2013). Age-related changes in the somatosensory processing of tactile stimulation: An fMRI study. Behav. Brain Res..

[25] Chapman L.J., Chapman J.P. (1987). The measurement of handedness. Brain Cogn..

[26] Futami R., Seki K., Kawanishi T., Sugiyama T., Cikajlo I., Handa Y. Application of local EMG-driven FES to incompletely paralyzed lower extremities. Proceedings of the 10th Annual Conference of IFESS.

[27] Tsuzuki D., Jurcak V., Singh A.K., Okamoto M., Watanabe E., Dan I. (2007). Virtual spatial registration of stand-alone fNIRS data to MNI space. NeuroImage.

[28] Jasper H.H. (1958). The ten–twenty electrode system of the International Federation. Electroencephalogr. Clin. Neurophysiol..

[29] Ye J.C., Tak S., Jang K.E., Jung J., Jang J. (2009). NIRS-SPM: Statistical parametric mapping for near-infrared spectroscopy. NeuroImage.

[30] McCormick P.W., Stewart M.S., Lewis G., Dujovny M., Ausman J.I. (1992). Intracerebral penetration of infrared light. J. Neurosurg..

[31] Murata Y., Sakatani K., Katayama Y., Fukaya C. (2002). Increase in focal concentration of deoxyhaemoglobin during neuronal activity in cerebral ischaemic patients. J. Neurol. Neurosurg. Psychiatry.

[32] Murkin J.M., Arango M. (2009). Near-infrared spectroscopy as an index of brain and tissue oxygenation. Br. J. Anaesth..

[33] Fujiwara N., Sakatani K., Katayama Y., Murata Y., Hoshino T., Fukaya C., Yamamoto T. (2004). Evoked-cerebral blood oxygenation changes in false-negative activations in BOLD contrast functional MRI of patients with brain tumors. NeuroImage.

[34] Seiyama A., Seki J., Tanabe H.C., Sase I., Takatsuki A., Miyauchi S., Eda H., Hayashi S., Imaruoka T., Iwakura T. (2004). Circulatory basis of fMRI signals: Relationship between changes in the hemodynamic parameters and BOLD signal intensity. NeuroImage.

[35] Schroeter M.L., Zysset S., von Cramon D.Y. (2004). Shortening intertrial intervals in event-related cognitive studies with near-infrared spectroscopy. NeuroImage.

[36] Kurth R., Villringer K., Curio G., Wolf K.J., Krause T., Repenthin J., Schwiemann J., Deuchert M., Villringer A. (2000). fMRI shows multiple somatotopic digit representations in human primary somatosensory cortex. Neuroreport.

[37] Schaefer M., Heinze H.J., Rotte M. (2005). Seeing the hand being touched modulates the primary somatosensory cortex. Neuroreport.

[38] Calvo-Merino B., Glaser D.E., Grèzes J., Passingham R.E., Haggard P. (2005). Action observation and acquired motor skills: An fMRI study with expert dancers. Cereb. Cortex.

[39] Buccino G., Lui F., Canessa N., Patteri I., Lagravinese G., Benuzzi F., Porro C.A., Rizzolatti G. (2004). Neural circuits involved in the recognition of actions performed by nonconspecifics: An fMRI study. J. Cogn. Neurosci..

[40] Craighero L., Mele S., Gaifas V., Bonaguri E., Straudi S. (2023). Evidence of Motor Resonance in Stroke Patients with Severe Upper Limb Function Impairments. Cortex.

[41] Zhang Y., Zhang Y., Xing B., Li J., Yang C., Han C., Wang Q. (2019). Mirror therapy versus action observation therapy: Effects on excitability of the cerebral cortex in patients after strokes. Int. J. Clin. Exp. Med..

[42] Cui Y., Cong F., Huang F., Zeng M., Yan R. (2023). Cortical activation of neuromuscular electrical stimulation synchronized mirror neuron rehabilitation strategies: An fNIRS study. Front. Neurol..

[43] Guirro R.R.J., Guirro E.C.O., de Sousa N.T.A. (2015). Sensory and motor thresholds of transcutaneous electrical stimulation are influenced by gender and age. PM R.

